# Supplementary figures and images for: CEPR2 perceives group II CEPs to regulate cell surface receptor-mediated immunity in Arabidopsis
Source: PLoS Pathog. 2025 Sep 22;21(9):e1013115. doi: 10.1371/journal.ppat.1013115 (PMC12494267; doi:10.1371/journal.ppat.1013115)

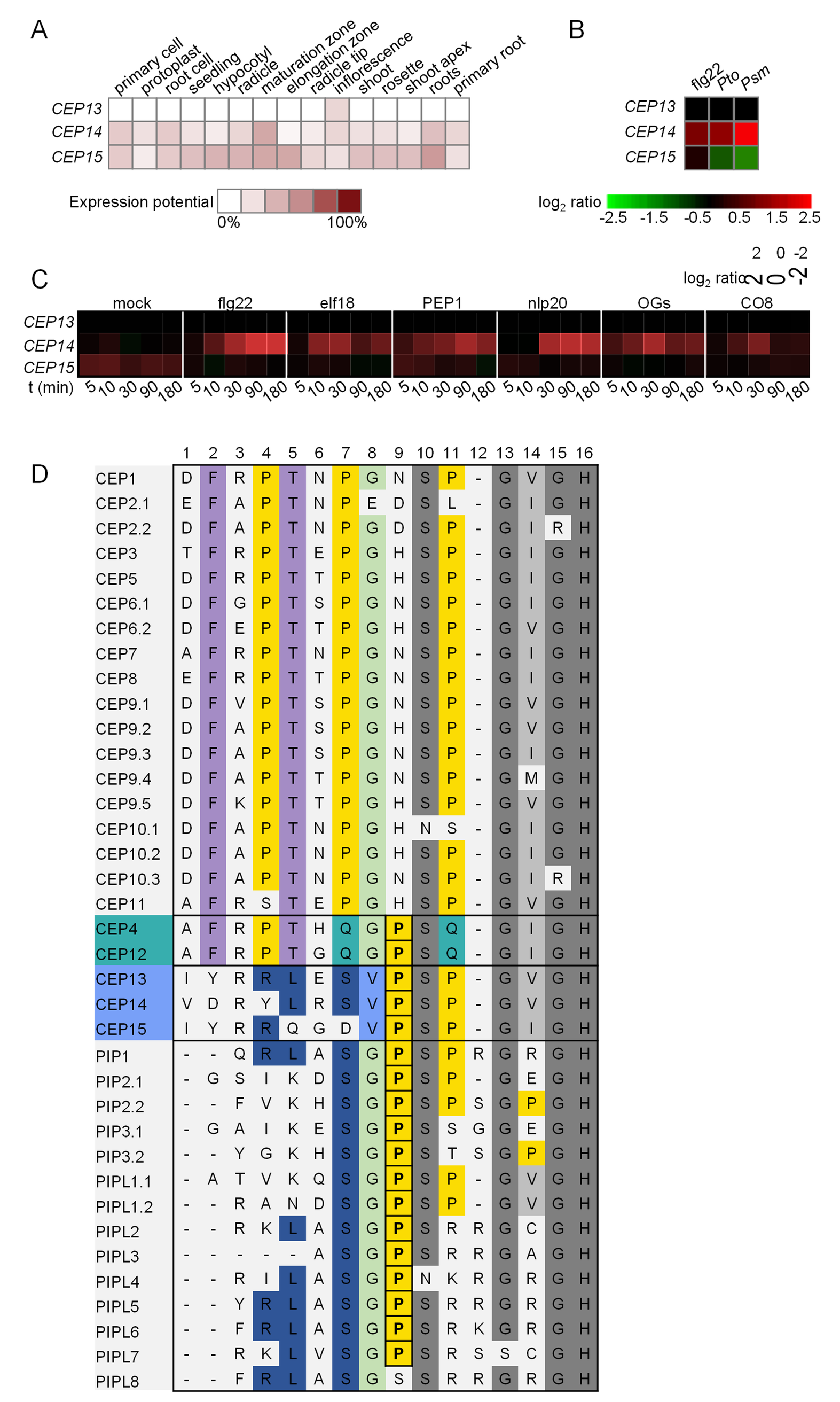

Supplement: S1 Fig — A) Tissue-specific expression potential of class II CEP family members. B) CEP expression after flg22 treatment and Pto/Pseudomonas syringae pv. maculicola (Psm) infection. C) Expression heat map of class II CEPs in wild-type seedlings following mock, flg22, elf18, PEP1, nlp20, OGs and CO8, treatment for the indicated time. Log2 (fold change) relative to time-point 0 h for each treatment. The data was obtained from Bjornson et al., 2021 [14]. D) Comparison of CEP and PIP putative mature peptide sequences aligned with the MAFFT tool. Conserved sequence features are highlighted to illustrate similarities and distinctions among the families. The C-terminal GxGH motif and Ser10 are shaded in dark grey, with the variable “x” position among CEPs and PIPs shown in light grey. Specific residues are color-coded for clarity: all proline residues are highlighted in yellow, CEP4/CEP12-specific residues in turquoise, class II CEP-specific residues in light blue, and class I-specific residues are shown in purple. Residues shared between certain class II CEPs and PIPs are highlighted in dark blue, and residues shared between class I CEPs and PIP/PIPLs are highlighted in light green. The proline at position 9, which is present in CEP4, CEP12, CEP13, CEP14, CEP15, and PIP/PIPLs but absent in canonical class I CEPs, is highlighted in bold and framed. Data in A and B were obtained using Genevestigator software and are based on the AT_mRNASeq_ARABI_GL-1 data set. (TIF) [file ppat.1013115.s001.tif]

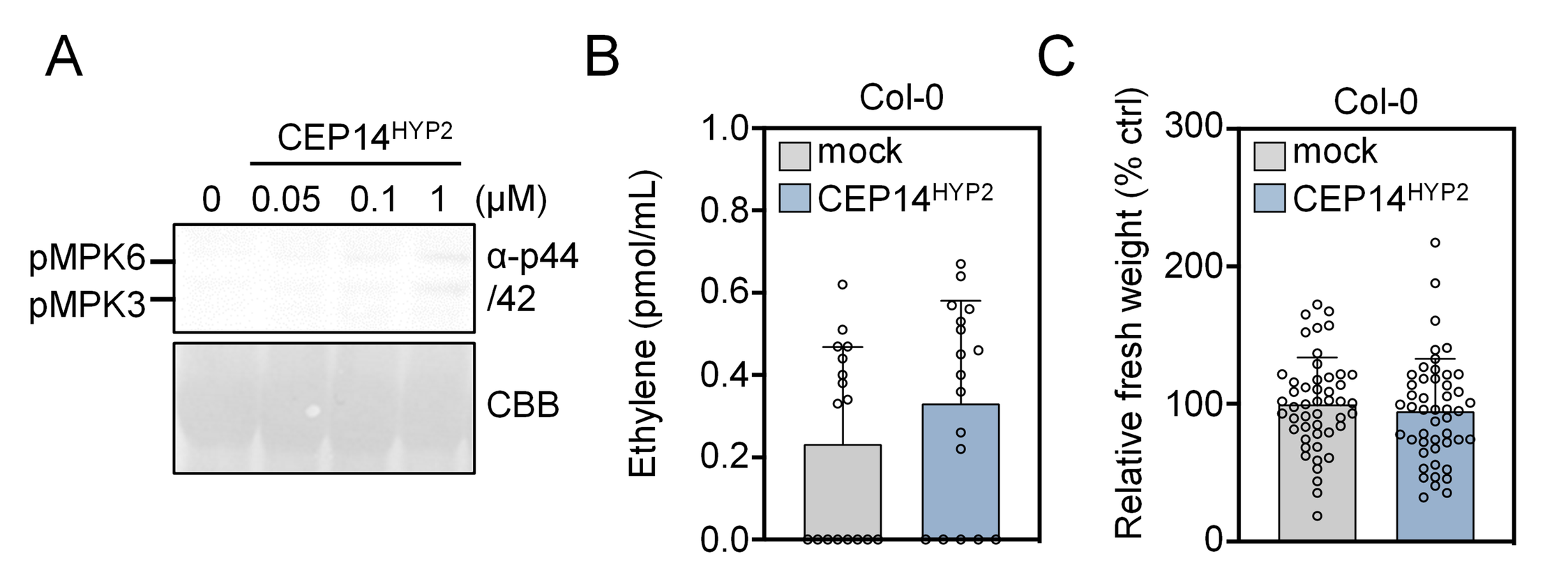

Supplement: S2 Fig — Western blots were probed with α-p44/42. CBB = Coomassie brilliant blue. B) Ethylene accumulation in Col-0 leaf discs 3.5 h upon mock (ddH2O) or CEP14HYP2 (1 μM) treatment; n = 17 pooled from four independent experiments, with mean ± SD (Mann-Whitney test, p = 0.2012). C) Relative fresh weight of five-day-old seedlings treated with CEP14HYP2 (1 μM) for seven days; n = 48 pooled from four independent experiments, with mean ± SD (two-tailed Student’s t-test, p = 0.4974). All experiments were performed at least three times in independent biological replicates with similar results. (TIF) [file ppat.1013115.s002.tif]

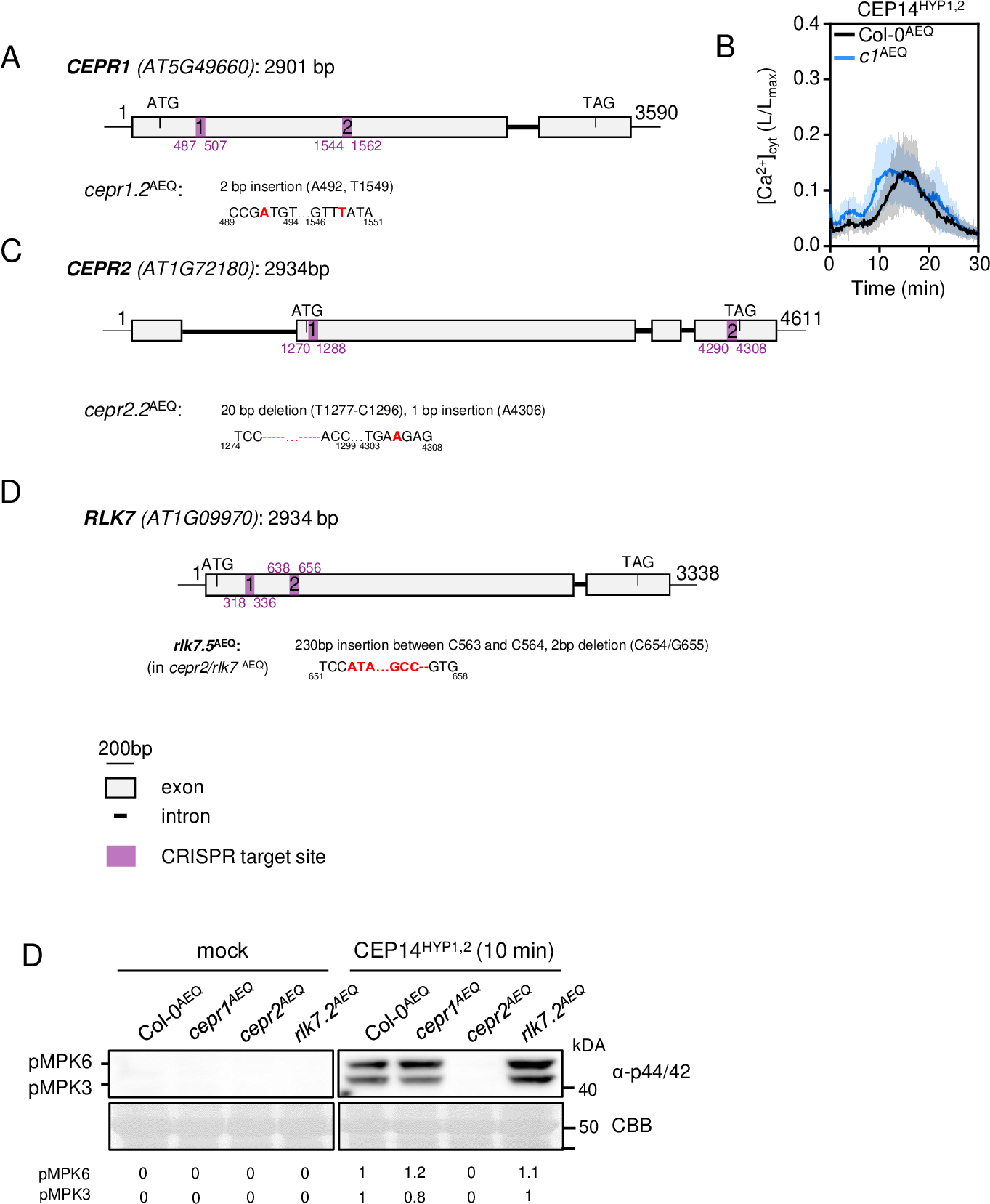

Supplement: S3 Fig — Schematic diagram of the CEPR1 (A), CEPR2 (B) and RLK7 (C) genomic sequence, structure, and the CRISPR-Cas9-mediated mutation pattern detected by DNA sequencing. The locus number and the length of the coding sequence (CDS) are indicated above the scheme for each gene. The CRISPR cepr1AEQ single mutant (with CRISPR allele cepr1.2AEQ) was generated in Col-0AEQ background with a CRISPR cepr1 construct. The CRISPR cepr2.2AEQ mutant was generated with a construct targeting both CEPR1 and CEPR2 [12]. The cepr2 rlk7AEQ line was generated in the cepr2.2AEQ background with a previously described rlk7 construct [12]. The specific location and type of mutations for each gene are indicated in the schematics describing the mutants. The two CRISPR target sites are indicated in purple, exons are indicated in grey, and introns are shown as black lines; scale bar = 200 bp. D) MAPK activation in indicated genotypes 10 min upon CEP14HYP1,2 treatment (100 nM). Western blots were probed with α-p44/42. CBB = Coomassie brilliant blue. Band intensities of pMPK6 and pMPK3 were quantified and normalized to the Rubisco band (CBB stain) for each lane relative to Col-0AEQ CEP14Hyp1,2 treatment. (TIF) [file ppat.1013115.s003.tif]

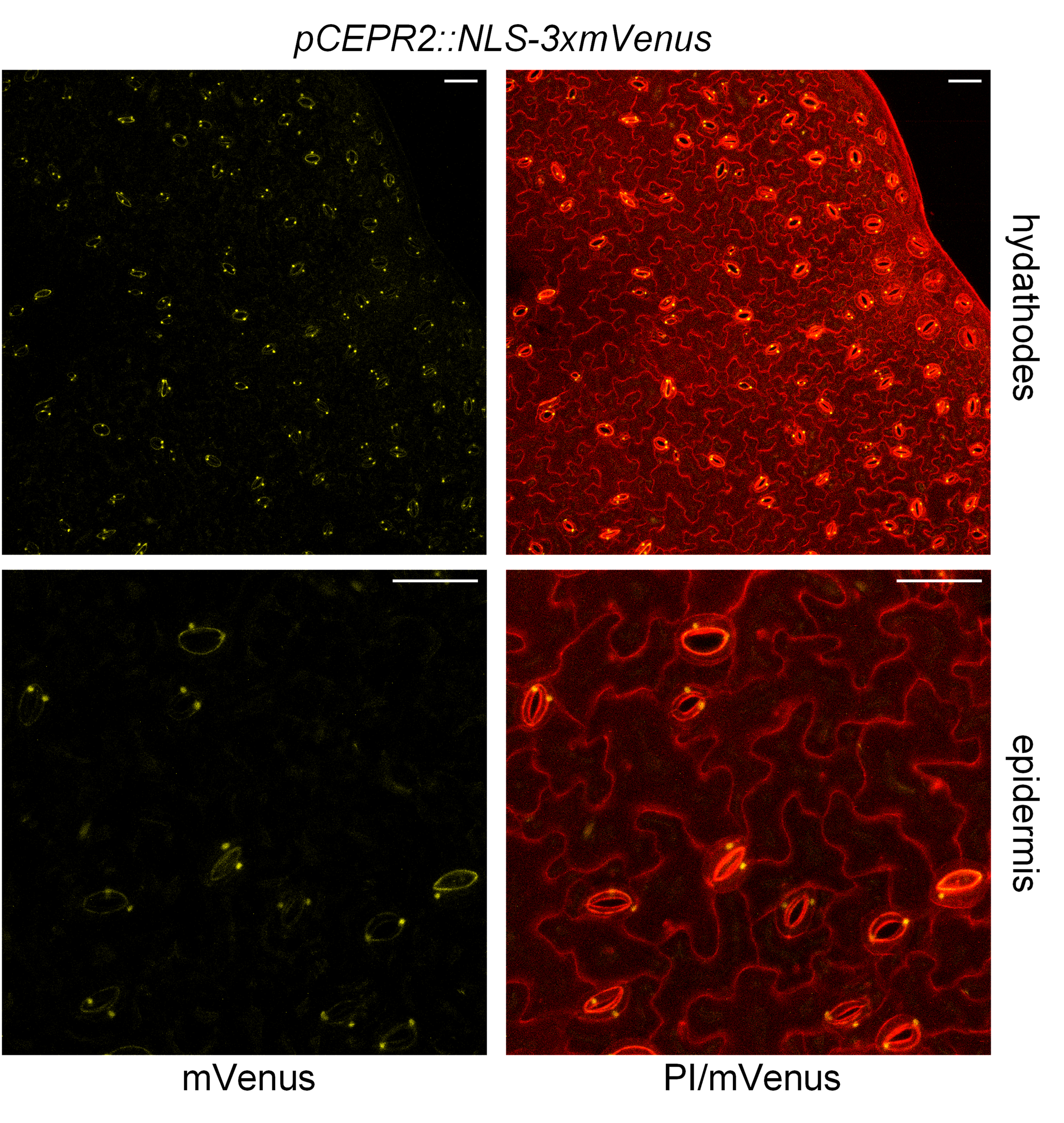

Supplement: S4 Fig — Representative images of NLS-3xmVenus signal in pCEPR2::NLS-3xmVenus lines around hydathodes and the epidermis. The maximum projection of Z-stacks for mVenus is merged with the Z-stacked PI signal; scale bar = 50 μm. The experiment was performed three times in independent biological repeats with similar results. (TIF) [file ppat.1013115.s004.tif]

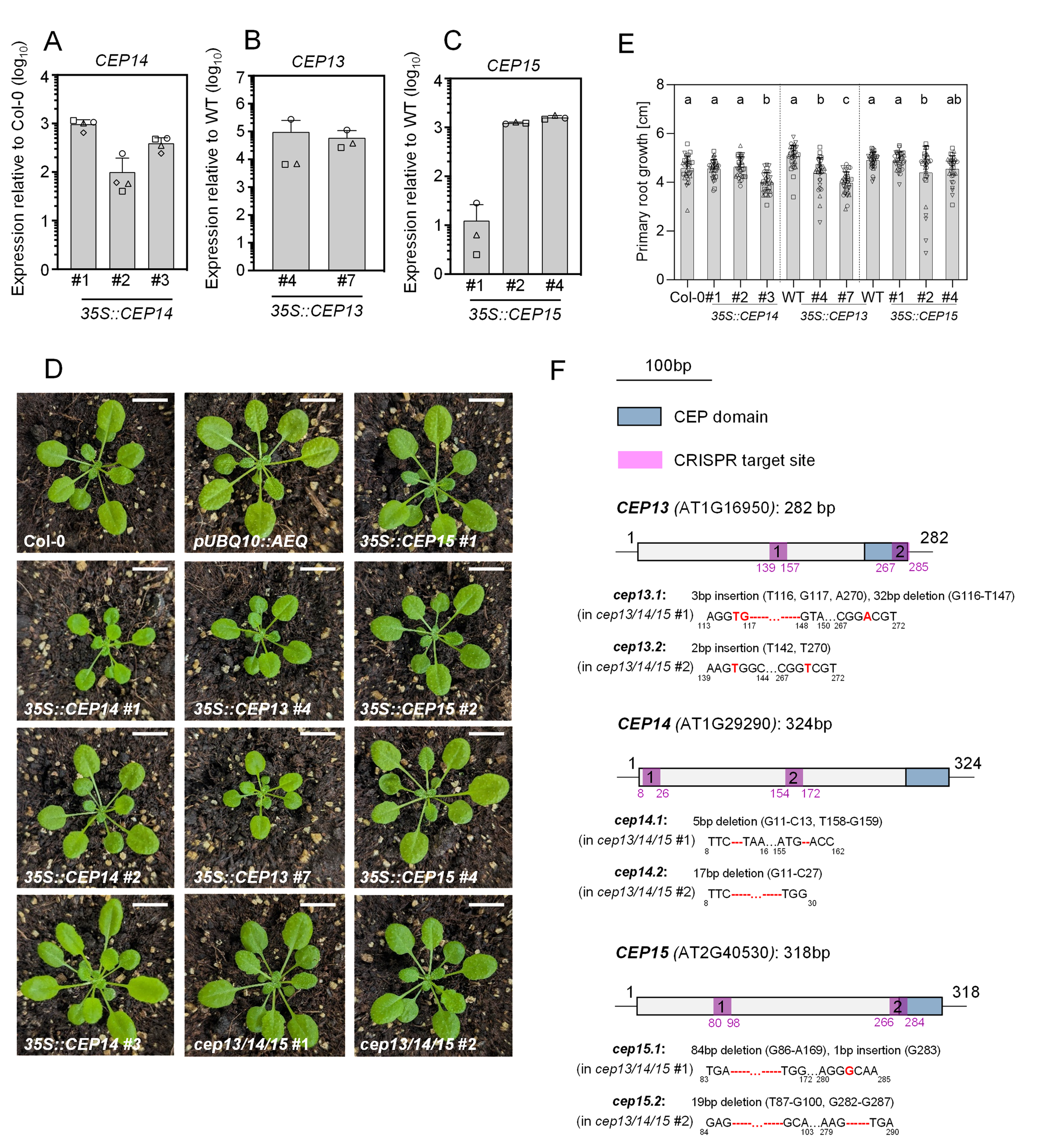

Supplement: S5 Fig — A) CEP14 transcript levels in three independent CEP14 overexpression lines, shown as fold expression compared to Col-0. Housekeeping gene UBQ5; n = 4 with mean ± SD. B) CEP13 transcript levels in two independent CEP13 overexpression lines, shown as fold expression compared to wild-type (aequorin reporter line, pUBQ10::AEQ). Housekeeping gene UBQ5; n = 3 with mean ± SD. C) CEP15 transcript levels in three independent CEP15 overexpression lines, shown as fold induction compared to wild-type (aequorin reporter line, pUBQ10::AEQ). Housekeeping gene UBQ5; n = 3 with mean ± SD. D) Pictures of 5-week-old plants of the indicated genotypes grown on soil. Sale bar = 1 cm. E) Seedlings were grown on 1/2 MS phytagel plates under long-day conditions. Germinated seedlings were transferred to fresh plates and imaged after 5 days. The primary root length was measured using Fiji (ImageJ) with the NeuronJ plug-in. Data represent mean ± SD from n = 32–33 pooled from independent experiments. The dotted line indicates separate experiments and statistical comparisons (one-way ANOVA, Tukey post-hoc test for CEP14 lines a-b p < 0.0001; for CEP13 lines a-b p < 0.0001, a-c p < 0.0001, b-c, p = 0.0078; for CEP15 lines WT vs #2 a-b, p = 0.0144, #1 vs #2 p = 0.0159). F) Characterization of two independent cep13/14/15 mutants, CRISPR cep13 cep14 cep15 #1 with alleles cep13.1, cep14.1, cep15.1 and #2 mutant with alleles cep13.2, cep14.2, cep15.2. Schematic diagram of CEP13, CEP14 and CEP15 gene structure and the CRISPR-Cas9-mediated mutation pattern detected by DNA sequencing. The locus number and the length of the CDS are indicated above the scheme for each gene. The specific location and type of mutations for each gene are indicated in the schematics describing the mutants. The CEP domain is indicated in blue, and the two CRISPR target sites are indicated in purple. Scale bar = 100 bp. (TIF) [file ppat.1013115.s005.tif]

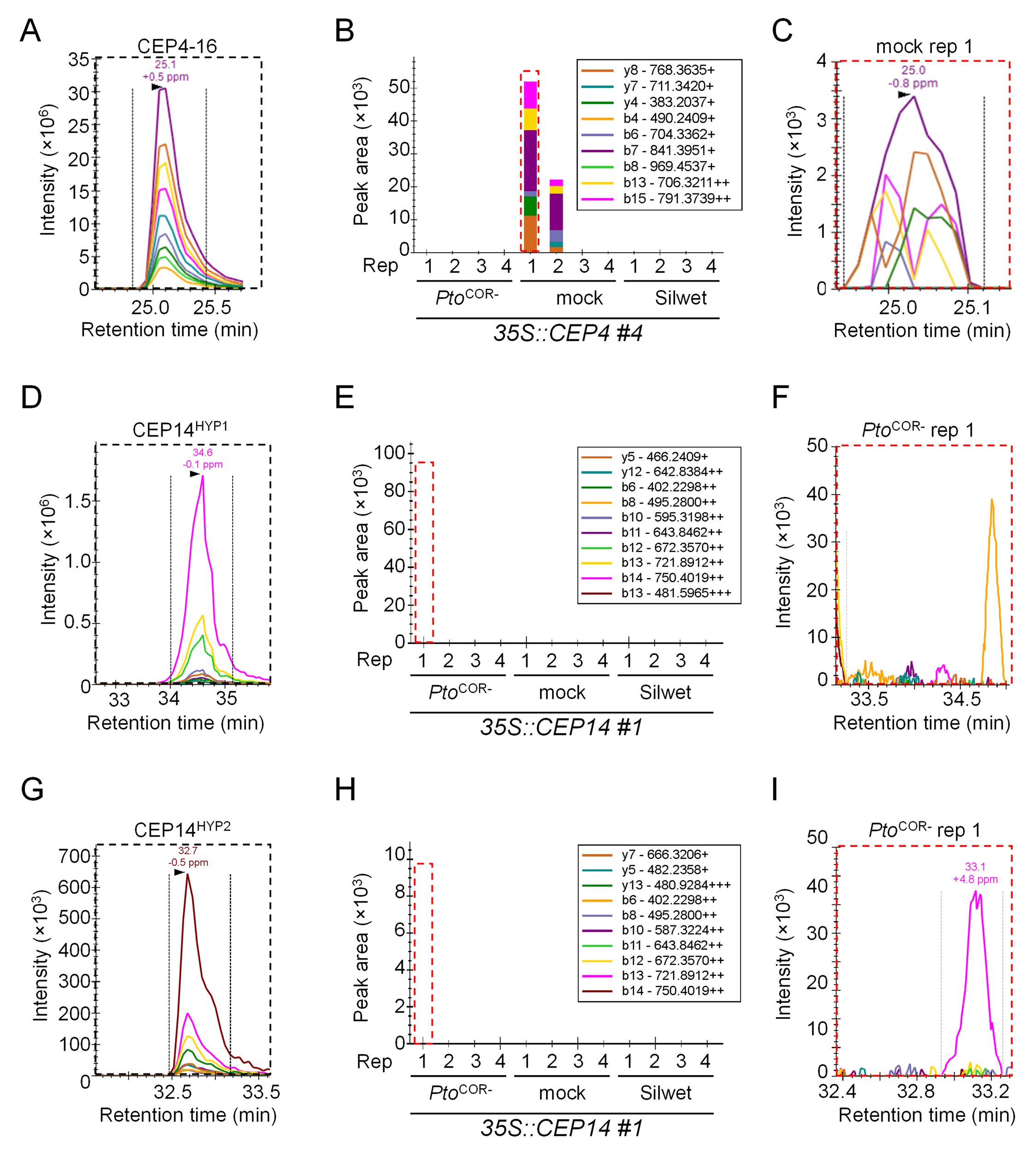

Supplement: S6 Fig — A) Extracted ion chromatograms of synthetic CEP416 peptide. B) Quantification of CEP416 in AWFs from PtoCOR--treated, mock-treated and Silwet-treated 35S::CEP4 #4 plants (four biological replicates per condition). CEP416 was endogenously detectable in only two out of 12 tested samples. C) Exemplary data of CEP416 peptide detected in AWF from PtoCOR--treated 35S::CEP4 #4 rep 1 plants. D) Extracted ion chromatograms of synthetic CEP14HYP1 peptide. E) Quantification of CEP14HYP1 in AWFs from PtoCOR--treated, mock-treated and Silwet-treated 35S::CEP14 #1 plants (four biological replicates per condition). CEP14HYP1 was not endogenously detectable. F) Exemplary data of endogenous CEP14HYP1 in AWFs from PtoCOR--treated 35S::CEP14 #1 rep 1 plants. G) Extracted ion chromatograms of synthetic CEP14HYP2 peptide. H) Quantification of CEP14HYP2 in AWFs from PtoCOR--treated, mock-treated and Silwet-treated 35S::CEP14 #1 plants (four biological replicates per condition). CEP14HYP2 was not endogenously detectable. I) Exemplary data of endogenous CEP14HYP2 in AWFs from PtoCOR--treated 35S::CEP14 #1 rep 1 plants. All synthetic peptides were measured at a concentration of 450 fmol using Parallel Reaction Monitoring. The dotted red squares in B, E and H highlight the chromatogram examples shown in C, F and I, respectively. The area under the curve of each coloured chromatogram reflects the MS intensity of a specific fragment ion. The sum of the 10 most intense fragment ions reflects the peptide MS intensity. (TIF) [file ppat.1013115.s006.tif]
