# Supplementary material for: CEPR2 perceives group II CEPs to regulate cell surface receptor-mediated immunity in Arabidopsis
Source: PLoS Pathog. 2025 Sep 22;21(9):e1013115. doi: 10.1371/journal.ppat.1013115 (PMC12494267; doi:10.1371/journal.ppat.1013115)
Supplement: Attachment — Submitted filename: !250704_response to reviewers.docx [file ppat.1013115.s011.docx]

**Reviewers' Comments:**

Reviewer's Responses to Questions

**Part I - Summary**
Please use this section to discuss strengths/weaknesses of study, novelty/significance, general execution and scholarship.

Reviewer #1: In this study, the authors investigated the role of group II CEPs and their receptor CEPR2 to regulate cell surface immunity in Arabidopsis. Overall, this is a well presented body of work with clear evidence to support the conclusion. The findings are interesting and provide valuable information for researchers working on plant-pathogen interaction. However, some issues should be addressed before it accepted for publication on PLoS Pathogens.

>>> Thank you very much for the positive assessment of our work. We appreciate the constructive feedback and will carefully address all raised points in the revised manuscript.

Reviewer #2: This manuscript by Rzemieniewski et al. demonstrated the group II CEP peptides CEP14, CEP13, and CEP15 act as phytocytokines to trigger hallmark immune responses and contribute to plant resistance against phytobacteria. The perception of group II CEPs is mainly dependent on CEPR2. I found the main conclusion of this MS is relatively similar with the recently published work at Plant Physiology (Wang et al, Plant Physiology, 2025). Although this study extended the analysis to show CEP13 and CEP15 are also able to induce immunity, their functional peptides are relatively conserved and exhibit the similar mode of action with CEP14. In addition, RLK7 is shown to participate in the CEP14/CEPR2-mediated Ca2+ influx, how it is implicated in the receptor complex and specific for Ca2+ response are not determined.

>>> Thank you very much for the positive assessment of our work. While we acknowledge conceptual overlap with Wang et al., *Plant Physiology*, 2024, our study was submitted within the 6-month scoop protection period and provides additional functional evidence for CEP13 and CEP15 activity and CEPR2 dependency. We appreciate the constructive feedback and will carefully address all raised points in the revised manuscript.

Reviewer #3: In this manuscript, Rzemieniewski et al., present research on the role of group II CEPs in plant immunity, specifically focusing on CEP14 and its receptor CEPR2 in Arabidopsis. The study provides evidence that group II CEPs are involved in PTI-like responses, contribute to antibacterial resistance, and that CEPR2 is required for the CEP14 recognition. The results are presented in a concise manner, and they support the primary conclusions. Meanwhile, I have several comments that should be addressed in the revised manuscript. The following points outline these comments

>>> Thank you very much for the positive assessment of our work. We appreciate the constructive feedback and believe that we could now address all raised points in the revised manuscript.

**Part II – Major Issues: Key Experiments Required for Acceptance**
Please use this section to detail the key new experiments or modifications of existing experiments that should be absolutely required to validate study conclusions.

Generally, there should be no more than 3 such required experiments or major modifications for a "Major Revision" recommendation. If more than 3 experiments are necessary to validate the study conclusions, then you are encouraged to recommend "Reject".

Reviewer #1:

1. Does CEPs, such as CEP14 specifically induced by flag22 and Pto? What about fungal pathogens or fungal PAMPs?

>>> Based on published transcriptomic data (Bjornson et al., *Nature Plants* 2021), which we reanalyzed and included in our manuscript, *CEP14* expression is also upregulated in response to chitin (CO8), a fungal PAMP. This data is depicted in Fig. S1C.

2. How CEPR2 perceives CEP13, CEP14 and CEP15? Via interaction or other ways? Please provide evidence.

>>> We thank the reviewer for this question. Our genetic evidence demonstrates that CEP13-, CEP14-, and CEP15-induced PTI responses are abolished (e.g., MAPK activation) or strongly reduced (e.g., calcium influx data shown for CEP14) in *cepr2* loss-of-function mutants, indicating functional dependency on CEPR2. CEP13, CEP14 and CEP15 are secreted peptides, and interaction between CEP14 and CEPR2 was previously demonstrated by co-immunoprecipitation and binding assays (Wang et al., *Plant Physiology* 2024). Furthermore, direct binding of other CEP family members to the extracellular domain of CEPR2 and related LRR-RLKs has been reported by us and others (Tabata et al., *Science* 2014, Rzemieniewski et al., *Nature Communications* 2024), supporting a conserved mechanism of ligand-receptor recognition via the extracellular domain in the apoplastic space. We now put more emphasis on these recent findings that support direct CEP14 perception by CEPR2 in the revised discussion section of our manuscript.

3. How different groups of CEPs perceive different receptors?

>>> We agree that this is an important and intriguing question. The spatiotemporal specificity of CEP ligand perception and how different CEPs engage distinct receptors or receptor complexes remains an open area of investigation. Notably, CEPs share sequence similarity with other signaling peptides such as PIPs , which are also perceived by the closely-related RLK7, suggesting a potentially conserved but nuanced recognition mechanism. While our current study focuses on the functional outputs of group II CEPs and their dependency on CEPR2, we recognize that the mechanistic basis of receptor specificity among CEP subgroups is not well understood and an exciting future research direction, which we also discussed in our manuscript.

4. Can you detect hydroxylated CEP14HYP2 in plant during pathogen infection? Or it can only be synthesized in vitro?

>>> Thank you for this question. Unfortunately, we were unable to detect CEP14^HYP1^, CEP14^HYP1,2^, CEP14^HYP2,^ or the non-hydroxylated CEP14 peptide in Arabidopsis apoplastic wash fluids under any of the tested conditions, including during pathogen infection. The exact reason for this remains unclear, but we suspect it may be due to technical limitations in mass spectrometry sensitivity or non-anticipated biochemical modifications of the CEP14 peptide precursors *in planta* that could interfere with detection, such as arabinosylation, which has been reported before for some group I CEP variants (Patel et al., 2018, Mohd-Radzman et al., 2015). We acknowledge the importance of this observation and elaborated on the possibility of additional PTMs in the revised discussion.

5. Figure 2B-C, likely missed a CEP14 control

>>> It is correct that we have not yet tested the non-hydroxylated CEP14 peptide in our assays. However, Wang et al. (*Plant Physiology*, 2024) have shown that the non-hydroxylated version of CEP14 is inactive in inducing MAPK activation, supporting the notion that hydroxylation is required for its biological function.

6. Figure 5, please display the inoculated plants, I want to see the phenotype.

>>> We thank the reviewer for this suggestion. Unfortunately, we did not take pictures of the inoculated plants used for cfu quantification. Additionally, *Pto*^COR-^ is a hypovirulent strain, and infections usually do not result in strong visible disease symptoms at three days post inoculation, which is the time-point used for bacterial isolation. Furthermore, we have to harvest the majority of plant material for bacterial isolation and cfu counting, which does not allow us to keep plants for an extended period for disease assessment by photography.

7. The CEPs-CEPR recognition mode is conserved in plant or not?

>>> CEPR2 is conserved in all seed plants. Group II CEPs are relatively under-characterized but are predicted to be conserved among angiosperm genomes (Delay et al., *Journal of Experimental Botany* 2013; Furumizu et al., *Plant Cell* 2021).

Reviewer #2: (No Response)

Reviewer #3: Major comments:
1. Lack of Direct Evidence for CEP14-CEPR2 Interaction: While the genetic and functional data suggest that CEPR2 is a key receptor for CEP14, there is no direct evidence of their physical interaction.

>>> We thank the reviewer for raising this important point. As outlined in our response to Reviewer #1, our genetic data provide evidence that CEP13-, CEP14-, and CEP15-induced PTI outputs are abolished (e.g., MAPK activation) or strongly reduced (e.g., calcium influx data shown for CEP14) in *cepr2* loss-of-function mutants. These findings indicate that CEPR2 is functionally required for the immune activity of all three group II CEP peptides.

CEP13, CEP14 and CEP15 are secreted peptides, and interaction between CEP14 and CEPR2 was previously demonstrated through co-immunoprecipitation assays (Wang et al., *Plant Physiology* 2024). Additionally, direct binding of other CEP family members to CEPR2 and related receptors has been shown in our previous work and others (Tabata et al., *Science* 2014, Rzemieniewski et al., *Nature Communications* 2024). Based on these genetic dependencies and published binding studies, we conclude that group II CEP likely binds CEPR2 directly via its extracellular domain in the apoplast. While we agree that measuring binding affinities by biophysical and biochemical methods would further strengthen this conclusion, such experiments are beyond the scope of the current manuscript. We put more emphasis on these previous findings in our revised discussion.

2. Line 223 states that CEP14 functions as a positive regulator of immunity against leaf-infecting pathogens, based on Pto COR- infection data. This is a significant claim that requires broader support. Given that Pto COR- is a lab-modified pathogen, it would be important to assess the effect of CEP14 overexpression against wild-type Pto, Psm, and potentially fungal pathogens to validate this claim more comprehensively.

>>> We appreciate this critical observation. We agree that the original statement in line 223: *“This suggests that CEP14 functions as a positive regulator of immunity against leaf infecting pathogens”* may overstate the generality of our findings. However, it is important to emphasize that *Pto*^COR-^ is routinely used to assess outputs related to pattern-triggered immunity and the restriction of stomatal entry of the pathogen (e.g. Stegmann et al., *Science* 2017, George et al., *JBC* 2023, Hou et al., *Plant Signaling and Behavior* 2019). We rephrased our conclusions to avoid over-interpretation of our data in the revised results section.

3. The authors conclude that CEP14 plays a "particularly prominent role" in cell surface receptor-mediated immunity (lines 237-238). However, the data supporting this conclusion is not sufficiently robust. The PtoCOR- infection assays lack crucial controls. Specifically, the manuscript needs to include the analysis of cep14 single mutants alongside the cep13 and cep15 single mutants. The claim about CEP14's prominent role cannot be fully supported without a direct, side-by-side comparison of these single mutants and their corresponding phenotypes.

>>> We thank the reviewer for this important comment. Recently published evidence demonstrated that *cep14* single mutants do not exhibits defects in resistance to *Pto* infection, suggesting genetic redundancy with other group II CEP members. This is in line with previous reports on group I CEP members, which were shown to act redundantly in controlling immunity against spray-infected bacteria (Rzemieniewski et al., *Nature Communications* 2024).

In our study, all analyzed *CEP14* overexpression lines, two of three *CEP15* overexpression lines, and none of the *CEP13* overexpression lines displayed enhanced resistance to *Pto*^COR-^ infection (Fig. 5A, B). Most lines show comparable overexpression levels (Fig. S5A-C), except *35S::CEP15* #1, which did not show a statistically significant spray infection phenotype and also displayed lower expression. We thus agree with the reviewer that our original statement regarding a „particularly prominent role“ of CEP14 was not fully justified. The available data rather suggest that CEP14 and CEP15 may play a more prominent roles in bacterial resistance compared to CEP13. We rephrased our conclusions in the revised results section.

4. Through the manuscript, different Statistics analysis were used. Why? Please keep it consistent. For example, Line 836-840. Both one-way ANOVA, Tukey post-hoc test and Brown-Forsythe and Welch ANOVA test, Dunnett’s T3 post-hoc test were used in one panel figure.

>>> We thank the reviewer for pointing this out. The use of different statistical tests within the same figure was intentional based on rigorous testing of variance and normality assumptions for each dataset. Specifically, for each dataset, we tested normality using the Shapiro-Wilk test and QQ plot analysis, and homogeneity of variance was tested using the F test (for paired analyses) or the Brown-Forsythe test (for multiple groups). When both assumptions were met, we used one-way ANOVA with Tukey’s post-hoc test. For datasets that failed the variance assumption (but passed normality), we applied Welch’s ANOVA with Dunnett’s T3 post-hoc test. In cases where the data was non-Gaussian, we used non-parametric tests such as the Kruskal-Wallis test with Dunn’s multiple comparisons. This approach ensures that the most appropriate and statistically valid method was chosen for each comparison. In figure 5B, for example, different statistical tests were used for *35S:CEP13*-Col-0 and *35S:CEP15*-Col-0 datasets because the variance assumptions differed between them. To improve clarity, we will explicitly state the rationale for using different tests in the revised *Methods: Statistics and reproducibility* section.

5. Figure 5, 35s:CEP14 plants showed enhance resistance to PtoCOR-, whether this phenotype depends on CEPR2?

>>> We agree that this is an interesting experiment. However, we do not have the *35S::CEP14* overexpression lines in a *cepr2* mutant background, and generating such genetic material would require considerable time beyond the suggested revision time frame. Our existing genetic and physiological data already indicate that CEPR2 is required for CEP14-triggered immune responses, including MAPK activation and calcium influx.

6. The authors' inability to detect CEP14 peptides in apoplastic wash fluids (AWFs) is a significant discrepancy that needs to be thoroughly addressed. This finding raises questions about the localization and mode of action of CEP14 and potentially weakens the overall conclusions. The authors must provide further experimental evidence or compelling explanations to resolve this issue.

>>> We appreciate the reviewer’s attention to this point and agree that the inability to detect CEP14 peptide in AWFs is both an interesting and important finding, and we acknowledge that it may raise some controversy. However, we deliberately decided to include this data in the manuscript because it emphasizes a broader issue in the field - namely, the need to confirm the identity of bioactive peptides *in planta*, which in most cases remain unknown. We elaborated more on this part in our revised discussion section. We do not think that localization of CEP14 secretion is a possible reason for the lack of *in vivo* peptide detection because we deliberately searched in *35S::CEP14* overexpression lines that should drive *CEP14* expression and secretion in most tissues.

**Part III – Minor Issues: Editorial and Data Presentation Modifications**
Please use this section for editorial suggestions as well as relatively minor modifications of existing data that would enhance clarity.

Reviewer #1: /

Reviewer #2: 1.The authors examined the requirement of hydrolation in the two proline residues of CEP14 to regulate immunity. Does the hydroxylation show selectivity in planta at these two tested sites ?

>>> We thank the reviewer for this interesting question. In a revised version of Figure 3, we now tested all hydroxylated CEP14 variants for their CEPR2 and/or RLK7 dependency. We conclude that irrespective of the hydroxylation pattern, *cepr2* mutants display strongly reduced and almost abolished calcium influx upon CEP14 treatments. The response is fully abolished in *cepr2 rlk7* double mutants which we recently generated (new Fig. S3D). We could not reproduce the reduced Calcium influx response previously observed for *rlk7* single mutants after moving the lab to a new location, nevertheless, given the fact that further mutation of RLK7 is required to fully abolish CEPR2-dependent CEP14 calcium influx, a minor role for RLK7 is still valid. We rephrased our discussion accordingly.

2.Fig 3A. The labeling of AEQ plants is not consistent in 3A and 3B. To rule out the dependency of CEPR1 in CEP14-induced immunity, the authors need to test the Ca2+ influx in cepr2/rlk7 to see whether the Ca2+ in completely block.

>>> We thank the reviewer for spotting the inconsistency between Fig. 3A and B, which we corrected in the revised version of the manuscript. In new experiments we noticed that the reduction in CEP14^Hyp1,2^, CEP14^Hyp2^ and CEP14^Hyp1^-triggered calcium influx was markedly stronger reduced in *cepr2*^AEQ^ mutants. A minor response was still recorded and completely blocked in the *cepr2 rlk7*^AEQ^ double mutants. However, the reduced response of *rlk7* to CEP14^Hyp1,2^-triggered calcium was no longer recorded in several repeats after re-establishing the assay in a new lab. We include the new data in a revised Fig. 3A.

3.Figure 5B Over-expression of CEP15 could enhance plant resistance to Ptocor-, did the authors try the protection assay by infiltrate CEP15 peptide followed by the infection.

>>> We thank the reviewer for this suggestion. We have not performed the protection assay via CEP15 peptide infiltration, as our study focuses on genetic approaches to dissect the role of CEP15 in bacterial resistance. Specifically, we demonstrate the contribution of CEP15 through overexpression analysis, and the combined role of group II CEPs in *Pto*^COR-^resistance using CRISPR-generated *cep13/14/15* triple mutants, which showed compromised resistance. We believe that our combined gain- and loss-of-function genetic data provide robust and physiologically relevant demonstration of CEP15’s contribution to immunity against *Pto^COR^*^-^.

4.What is the root phenotype in these CEP OE plants? The authors performed the growth inhibition by CEP14 treatment as the marker for immune activation, given that CEPs regulate root growth and architecture, will this growth inhibition be a complex output of growth and defense pathways?

>>> We thank the reviewer for this question. We agree that the observed reduction in seedling fresh weight may partially reflect an integration of developmental and immune signaling. We now tested whether *CEP13*, *CEP14* and *CEP15* overexpression may affect primary root elongation. We observed that both CEP13 overexpression lines displayed reduced primary root growth, while only one of three lines each for *35S::CEP14* and *35S::CEP15* displayed this phenotype. We included this data in the new Supplementary Fig. 5E.

Reviewer #3: Minor comments:
1. "Throughout the manuscript (e.g., lines 1, 17, 23, 45, 75, 79), the term 'cell surface immunity' is used. While the initial ligand-receptor recognition indeed occurs at the cell surface, the subsequent immune signaling cascade takes place within the cytosol and nucleus. To more accurately reflect this, consider rephrasing 'cell surface immunity' to 'cell surface receptor-mediated immunity' or similar

>>> We thank the reviewer for this helpful suggestion. In the revised manuscript, we will replace the term “cell surface immunity” with „cell surface receptor-mediated immunity.“

2. Line 869 pv. should not be italic.

>>> We thank the reviewer for spotting this error, which we corrected in the revised manuscript.

3. The presentation of data in Figure 3A and 3B needs improvement to enhance readability. The current color scheme and scaling make it difficult to discern the differences between the genotypes being compared.

>>> We thank the reviewer for this valuable feedback. We changed the color scheme in the revised figure 3. We also merged Figure 3 A and B in a new Figure 3A with 3 subpanels.

4. Please provide densitometry analysis or other quantification methods to assess the levels of phosphorylated MAPK in all related figures. This quantification is essential for proper interpretation of the data.

>>> We thank the reviewer for this suggestion. We now provide relative MAPK intensity quantifications in the revised manuscript.
